# Portrait of Tissue-Specific Coexpression Networks of Noncoding RNAs (miRNA and lncRNA) and mRNAs in Normal Tissues

**DOI:** 10.1155/2019/9029351

**Published:** 2019-09-03

**Authors:** Claudia Cava, Gloria Bertoli, Isabella Castiglioni

**Affiliations:** Institute of Molecular Bioimaging and Physiology, National Research Council (IBFM-CNR), Segrate (Mi), Italy

## Abstract

Genes that encode proteins playing a role in more than one biological process are frequently dependent on their tissue context, and human diseases result from the altered interplay of tissue- and cell-specific processes. In this work, we performed a computational approach that identifies tissue-specific co-expression networks by integrating miRNAs, long-non-coding RNAs, and mRNAs in more than eight thousands of human samples from thirty normal tissue types. Our analysis (1) shows that long-non coding RNAs and miRNAs have a high specificity, (2) confirms several known tissue-specific RNAs, and (3) identifies new tissue-specific co-expressed RNAs that are currently still not described in the literature. Some of these RNAs interact with known tissue-specific RNAs or are crucial in key cancer functions, suggesting that they are implicated in tissue specification or cell differentiation.

## 1. Introduction

The portion of the genome encoding proteins constitutes only 1.5% of the human genome, while the transcribed RNAs not yielding coding proteins, named non-coding RNAs (ncRNAs) [1], are the major part.

ncRNAs are capable of controlling the expression of many genes, thus can simultaneously control multiple cellular functions regulating a variety of physiological and pathological processes [2, 3].

The family of ncRNAs includes small ncRNAs such as miRNAs, piRNAs, and siRNAs and long ncRNAs (lncRNAs).

miRNAs, approximately 18–25 nucleotides in length, are highly conserved and involved in post-transcriptional gene silencing by binding complementary sequences in the 3′ untranslated region (3′ UTR) of messenger RNAs (mRNAs) [4]. Significant evidences in the last few years showed a fundamental role of miRNAs in determining cell fate, in pattern formation in embryonic development, and in controlling cell proliferation, cell differentiation, and cell death [4]. It has also been shown that aberrations in miRNA expression levels contribute to the onset and progression of many types of cancer [5–7].

Published studies have identified some tissue-specific (TS) or developmental-stage-specific miRNAs, suggesting their potential role in maintaining tissue identity and function [8–10]. The first comprehensive analyses of miRNAs across different human tissues have been reported in 2007, by Landgraf et al. [11] analyzing 340 distinct mature miRNAs in 26 human tissues and by Liang et al. [12] analyzing 345 distinct mature miRNAs in 40 human tissues. More recently, in 2016, Ludwig et al. profiled 1997 different mature miRNAs in 61 human tissues [13]. These studies showed that few miRNAs are expressed with a high degree of tissue specificity [11–13].

lncRNAs are transcribed RNA molecules bigger than 200 nucleotides in length, poorly conserved. They can regulate gene expression by different mechanisms that are not yet fully understood [3, 4]. Scientific evidences show they have roles in DNA replication, chromosomal dynamics, telomere biology, and subcellular structural organization [14–16].

Some works have found that lncRNAs tend to show even more TS expressions than mRNAs [14, 15]. For example, many lncRNAs are expressed only in limited developmental contexts or many of them are expressed during embryonic stem cell differentiation and in the brain (in a mouse model) [9]. However, few TS lncRNAs have been well characterized to date.

Although all human tissues carry out common processes, each tissue is characterized by specific gene expression patterns that are essential for the conservation and survival of the tissue environment in physiological conditions. According to the Human Protein Atlas [17], approximately 40% of human mRNAs show a higher gene level expression in one single tissue. However, these studies did not focus on TS-miRNAs, TS-lncRNAs, and TS-mRNAs interactions.

The common and TS processes are ultimately controlled by gene regulatory networks that can integrate the complex interactions between mRNAs, miRNAs, and lncRNAs. Since different tissues have different regulatory networks, mapping regulatory networks among TS-RNAs could represent a significant step towards the knowledge, on molecular basis, of tissues and on how tissue specificity is determined.

The most widely used network model is based on gene co-expression network [18, 19]. Co-expression networks of biological elements (TS-mRNAs, TS-miRNAs, and TS-lncRNAs) are interesting since co-expressed elements are regulated by the same, functionally related transcriptional program or by members of the same pathway or protein complex. However, most of the few published co-expression studies between mRNAs, miRNAs, and lncRNAs [11, 15, 20] are focused on a single tissue or on a small number of different tissues.

Furthermore, recent studies showed that those ncRNAs, proposed as cancer biomarkers, were not tissue specific or were not expressed in the tissues where the disease is supposed to originate [21, 22]. Thus, it is unexpected to think about these ncRNAs as cancer biomarkers specific for a tissue. Indeed, a robust cancer biomarker of a specific tissue should show enrichment in that tissue in which the tumor develops. These findings highlight the need of developing new computational models to identify disease-related miRNAs/lncRNAs based on the tissue-specific co-expression networks. For example, it is not sufficient to predict the association of some miRNAs to a disease only based on the similarity expression, but it is also necessary to include the tissue specificity of noncoding RNAs. Adding this analysis into several existing tools [23, 24] could lead to a more comprehensive knowledge and an improved precision in the prediction of the association between non-coding RNA and disease.

In our study, focusing on the co-expression network between TS-mRNAs, TS-miRNAs, and TS-lncRNAs, we generated a large overview of TS-distribution of RNAs by using thirty different human normal tissues and more than eight thousand human samples.

Our portrait may help to understand how genes, whose functions depend on their tissue context, are expressed and regulated in a normal condition.

## 2. Materials and Methods

### 2.1. Data

GTEx data [25] (2016 release) provided the expression levels of RNAs in thirty different human normal tissues: adipose tissue, adrenal gland, bladder, blood, blood vessel, brain, breast, cervix uteri, colon, esophagus, fallopian tube, heart, kidney, liver, lung, muscle, nerve, ovary, pancreas, pituitary, prostate, salivary gland, skin, small intestine, spleen, stomach, testis, thyroid, uterus, and vagina, specifically 1368 miRNAs, 10167 lncRNAs (as reported from lncRNome database [26]), and 30441 mRNAs (as reported from HUGO database [27]). The subjects enrolled for the study range from 6 to 1259 for a total of 8553 samples based on the examined tissue (Table 1). For each tissue, and for each sample, we filtered mRNA profiles by removing pseudogenes, and we calculated median values of RNA expression levels. We thus created an *m* × *N* expression matrix, being *m* the number of RNAs and *N* the number of tissues (1–30). As RNA expression levels, we considered the median values over all samples for that tissue. We removed RNAs with median expression values = 0.

### 2.2. Gini Index

The Gini index (GI) [28] was used to identify TS-RNAs (miRNAs, lncRNAs, and mRNAs). Being *m* the number of RNAs and *N* the number of tissues (1–30), GI is defined as

(1) $$\begin{array}{c} \frac{1}{N}\left(N+1-2\left(\frac{\sum_{j=1}^{N}\left(N+1-i\right)x_{ij}^{\prime }}{\sum_{j=1}^{N}x_{ij}^{\prime }}\right)\right), \end{array}$$

where *x*_*ij*_ is the median expression of the RNA *i* in tissue *j and x*_*i*_ is a vector in the nondescending order (*i* *=* *1*,…, *m*; *j* *=* *1*,…, *N*).

GI measures the specificity of expression of an RNA with respect to different tissues and ranges from 0 to 1 indicating RNAs expressed in many tissues (housekeepers and constitutive) for scores of GI close to 0, and RNAs expressed in only one specific tissue for scores of GI close to 1 [28].

### 2.3. Gini Index Selection of TS RNAs

We considered a TS-RNA *i* if GI*i* ≥ 0.85 and a nonspecific tissue RNA *i* if GI*i* ≤ 0.15 [13, 28]. For RNA *i* with GI*i* ≥ 0.85, we choose the TS where RNA expression profile has the highest value. Thus, an RNA can be TS only for one tissue but a tissue can have more TS RNAs.

### 2.4. Tissue-Specific miRNA-lncRNA-mRNA Regulatory Co-Expression Network

For each tissue, we generated a co-expression network: two RNAs are linked if their expression levels are correlated. We considered a co-expression interaction between two mRNAs if the Pearson correlation index was >0.7 or < −0.7. The correlation was calculated between expression levels of TS-miRNAs and TS-lncRNAs of the samples for each tissue. The same procedure was performed between TS-miRNAs and TS-mRNAs and between TS-mRNAs and TS-lncRNAs.

## 3. Results

### 3.1. Tissue-Specific RNAs

We present the results obtained from the Gini index used to measure tissue specificity.

**Figure 1:**
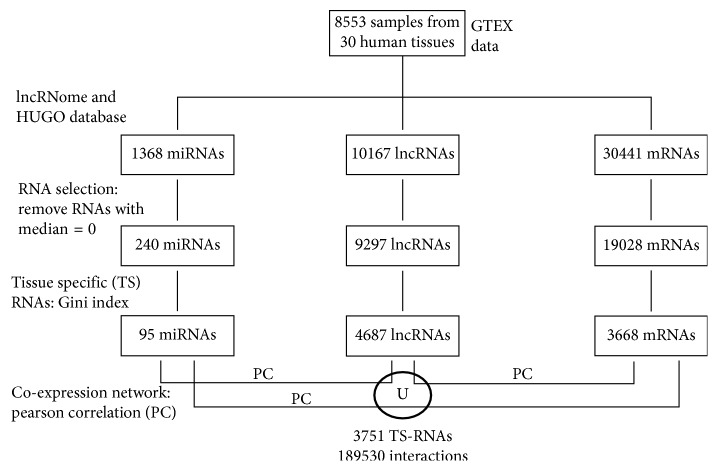
The flow chart of the whole data selection and results.

RNA selection leaded to 240 miRNAs, 9297 lncRNAs, and 19028 mRNAs. GI was calculated from these selected miRNas, lncRNAs, and mRNAs (Figure 1). Only 4 miRNAs (*miR-302b*, *miR-611*, *miR-1282*, and *miR-4721*) (1.6%) were ubiquitously expressed with a GI < 0.15. Ninety-five miRNAs (39.7%) showed a TS expression with GI > 0.85 (Figure 1). The largest group of all miRNAs (58.7%) have 0.15 < GI < 0.85.  Table 2 shows the partition of 95 miRNAs in the tissues.

No TS-miRNA was found for the adipose tissue, blood vessel, breast, colon, esophagus, heart, pancreas, stomach, uterus, and vagina. Testis contains the highest number of TS-miRNAs.

Only ∼1% lncRNAs were ubiquitously expressed with a GI < 0.15. The largest group (51%), including 4687 lncRNAs (Figure 1), showed a TS expression with GI > 0.85. However, 48.2% of all lncRNAs have 0.15 < GI < 0.85.

Table 3 shows the distribution of the 4687 lncRNAs for each tissue. TS-lncRNAs range from 1 in the esophagus to 3264 in the testis, representing the largest group. Supplementary [Supplementary-material supplementary-material-1] shows the list of TS-lncRNAs in detail.

About 9% mRNAs were ubiquitously expressed with GI < 0.15. A TS expression with GI > 0.85 was shown in 3668 mRNAs (Figure 1), and 72% of all mRNAs have 0.15 < GI < 0.85, representing the largest group.

Since the TS-mRNAs obtained from our analyses are only 19.3% of the total mRNAs, it appears that the regulation of the transcription by miRNAs and lncRNAs seems to be fundamental for the differentiation of each tissue.

Table 4 shows 3668 mRNAs for each tissue. TS-mRNAs range from 2 in the esophagus to 1563 in the testis, representing the largest group.

### 3.2. Tissue-Specific miRNA-LNC-mRNA Regulatory Networks

For each tissue type considered in this work, we analyzed the number of co-expression interactions between TS-miRNAs, TS-lncRNAs, and TS-mRNAs, and we present the results in Table 5.

The most numerous co-expression networks involve the testis, brain, small intestine, skin, and liver with 184041, 1512, 1197, 711, and 476 interactions, respectively. No TS-gene regulatory network was found in the blood vessel, esophagus, and uterus. In total, we found 189530 interactions and 3751 TS-RNAs (Figure 1).

The flow chart of the whole data selection and results is shown in Figure 1, for better synthesis of methodology.

## 4. Discussion

In the past decades, functional studies have focused on the analysis of protein-coding transcripts (mRNA expression) to characterize biological processes such as metabolism, cell differentiation, immune response, and tumorigenesis. The recent growth of next-generation sequencing technology has brought the discovery and characterization of a new class of non-coding RNA transcripts, such as miRNAs and lncRNAs. Evidences from high-throughput genomic platforms suggest the importance of ncRNAs in the regulation of multiple major biological processes such as cell development, differentiation, and metabolism [2].

However, despite successful studies highlighting important roles of miRNAs and lncRNAs in different tissues and diseases, no comprehensive portrait across various tissue types has been reported.

To assess the relation between mRNAs, miRNAs, and lncRNAs in several normal tissues, by using a computational approach, we investigated tissue specificity of miRNAs, lncRNAs, and mRNAs across 30 normal tissue types, obtaining 95 TS-miRNAs, 4687 TS-lncRNAs, and 3668 TS-mRNAs.

Our analysis on mRNAs suggests that they are not the determinants of tissue phenotype since only 19.3% of the total mRNAs is tissue specific. On the contrary, our study confirms the high specificity of lncRNAs in various tissues, consistently with previous studies [15, 29]. Indeed, we found that 51% of lncRNAs show high tissue specificity, highlighting the role of lncRNAs as regulators of gene expression in specific cellular phenotype. Thus, the identification and characterization of human lncRNAs with TS expression become crucial in order to determine their relevant functions.

Similarly to what is observed with lncRNAs, we found that 39.7% of miRNAs is tissue specific, also in this case proving the high tissue specificity of miRNAs and their critical role as regulators of mRNA expression, and thus, tissue phenotype.

We speculated that lncRNAs could (1) be controlled by miRNAs, reducing lncRNA stability and altering the cellular response in physiologic and pathologic processes; (2) act as endogenous sponges, harbouring similar microRNA target sequences and sequestering microRNAs; and (3) be precursors for the generation of miRNAs to silence target mRNAs, thus competing with endogenous miRNAs [30].

For these reasons, the exploration of the regulatory relationships between miRNAs, lncRNAs, and mRNAs, specifically expressed in the same tissue, could offer useful information to shed light on how miRNAs, lncRNAs, and mRNAs play a role in tissue specification or cell differentiation [31].

However, the limit of our work is that we focused our analysis on a single dataset for each considered normal tissue. Future studies should validate our results on an independent dataset. Furthermore, we did not assess the influence on gene expression of some risk factors, such as age, smoking status, gender, and body mass index; thus, further studies on datasets including phenotype risk data could improve our methodology and results.

Despite these limitations and although several studies found that non-coding RNAs have important roles in diverse physiology and disease processes, we proposed a unique study that comprehensively compared the expression levels of tissue-specific non-coding RNAs with those of tissue-specific mRNAs.

Since a great number of TS-mRNAs are also key biomarkers in specific cancer diseases, the identification of TS co-expression network could be useful to deepen TS function of RNAs at molecular basis of cancer.

### 4.1. Adipose Tissue

TS-co-expression gene regulatory network in adipose tissue involves 5 mRNAs (ADIPOQ, CIDEA, CIDEC, PLIN1, and TUSC5) and 1 lncRNA (ADIPOQ-AS1), sharing 5 interactions. Although all 5 mRNAs in previous studies have showed a principal role in adipose tissue, no study has revealed an interaction between them.

Adiponectin, C1Q and collagen domain containing (ADIPOQ) is expressed exclusively in adipose tissue and is involved in the control of fat metabolism and insulin sensitivity [32].

Cell death-inducing DFFA-like effector A (CIDEA), along with CIDEB and CIDEC, constitutes the CIDE family of proteins. They are important modulators of several lipid metabolic pathways, including lipolysis, fatty acid oxidation, VLDL lipidation, and lipid droplet growth in adipocytes and hepatocytes [33]. Perilipin 1 (PLIN1) is a modulator of adipocyte lipid metabolism, lipolysis, and triglyceride levels. It plays a role in lipid formation by regulating CIDEC. The CIDEC-PLIN1 interaction promotes lipid droplet proteins acting on fat metabolism in adipocytes [34].

Tumor suppressor candidate 5 (TUSC5) was found as an adipose TS protein that is abundantly expressed in white [35] and brown adipose tissue [36], and TUSC5 mRNA expression increases during adipogenesis [36].

The interactions between them involve a lncRNA, ADIPOQ-AS1, that shows a positive correlation with other mRNAs. ADIPOQ-AS1 is located within the genomic location of ADIPOQ.

### 4.2. Adrenal Gland

TS-co-expression gene regulatory network in the adrenal gland involves 6 mRNAs (CYP11A1, CYP11B1, FAM166B, MRAP, NR5A1, and VWA5B2) 2 lncRNAs (AP000266.7 and RP11-320G24.1) and 1 miRNA (*miR-1224*), sharing 6 interactions.

Cytochrome P450 family 11 subfamily A member 1 (CYP11A1), subfamily B member 1 (CYP11B1), and family with sequence similarity 166 member B (FAM166B) showed a high expression in the adrenal gland but no study demonstrated a particular role in the adrenal gland to date [17, 37].

Melanocortin 2 receptor accessory protein (MRAP) encodes a protein that controls protein trafficking and the functions of its receptor in the adrenal gland [38]. It also associated with adrenal disease and was detected in human adrenal and brain tissue [39].

Nuclear receptor subfamily 5 group A member 1 (NR5A1) is a nuclear receptor involved in gonadal and adrenal development. Mutations in this gene are a reason of XY sex reversal without a direct link with adrenal malfunctions [40].

von Willebrand factor A domain containing 5B2 (VWA5B2) was found expressed more than five fold in the human adrenal gland compared with other normal tissue types [41] but no study to date demonstrated a particular role in the adrenal gland. VWA5B2 showed a positive correlation with *miR-1124*.

AP000266.7 showed a positive correlation with MRAP and CYP11A1. AP000266.7 and MRAP are neighbor genes localized in chromosome 21.

RP11-320G24.1 showed a positive correlation with FAM166B, CYP11B1, and NR5A1. No relationship seems to be between them in literature.

### 4.3. Bladder

TS-co-expression gene regulatory network in the bladder involves 22 mRNAs (CLEC3A, CYP4F8, DHRS2, EVX1-AS, OR10H1, OR5M11, OR5P2, PADI3, PLA2G2F, S100P, SPINT4, TACR3, TMPRSS4, TRBV6-9, TRPA1, UGT1A1, UGT2B28, UPK1A, UPK1B, UPK2, UPK3A, and VGLL1), 25 lncRNAs (AC004543.2, AC009478.1, AC012307.2, AC017048.2, AC017060.1, AC019117.1, AC027119.1, AC079610.1, AC092669.3, AC097499.1, AC114812.8, AC114877.3, GS1-120K12.4, POU6F2-AS2, RP11-1078H9.2, RP11-109D24.1, RP11-113O24.3, RP11-128L5.1, RP11-272K23.3, RP11-463P17.1, RP11-753H16.4, RP11-875H7.5, RP11-91I8.1, RP3-405J10.4, and UPK1A-AS1), and 1 miRNA (*miR-3972*), sharing 199 interactions.

C-type lectin domain family 3 member A (CLEC3A) was originally identified as a cartilage protein from shark and bovine [42], but recently it was found for the first time to be associated to the urinary bladder in [43] along with dehydrogenase/reductase 2 (DHRS2) and peptidyl arginine deiminase 3 (PADI3).

Several of identified genes have been associated with the progression of bladder cancer, such as S100 calcium-binding protein P (S100P), belonging to a family of calcium-binding proteins, [44], tachykinin receptor 3 (TACR3) [45], and UDP glucuronosyltransferase family 1 member A1 (UGT1A1) [46].

Transient receptor potential cation channel subfamily A member 1 (TRPA1) is expressed in the urinary bladder [47] as well as uroplakins (UPK1A, UPK1B, UPK2, and UPK3A) with a role in regulating the membrane permeability [48].

Among lncRNAs, we found POU6F2-AS2, which showed a positive correlation with PADI3, UPK1B, S100P, TACR3, TRPA1, OR5P2, and TMPRSS4. Although POU6F2-AS2 has not yet been reported in the bladder, previous studies showed its role in the initiation and progression of esophageal squamous cell carcinoma [49]. No information has been reported in the literature about other lncRNAs.

### 4.4. Blood

TS-co-expression gene regulatory network in blood involves 27 mRNAs (APOBEC3A, AQP9, CSF3R, CST7, CXCR1, CXCR2, FCAR, FCGR3B, FFAR2, FPR1, FPR2, GYPA, KLF1, LILRA5, MMP9, NFE2, PADI4, PROK2, S100A9, S100A12, SELL, TRAV23DV6, TRAV9-2, TREM1, TREML2, VNN2, and VNN3), 11 lncRNAs (AC008984.2, AOAH-IT1, RP11-166N6.2, RP11-321E2.4, RP11-342D11.3, RP11-356C4.3, RP11-405F3.4, RP11-465L10.10, RP11-561P12.5, RP1-55C23.7, and TRBV11-2), and 2 miRNAs (*miR-144* and *miR-223*), sharing 96 interactions.

Apolipoprotein B MRNA editing enzyme catalytic subunit 3A (APOBEC3A) induces RNA editing in monocytes and macrophages [50]. Aquaporin 9 (AQP9) was found expressed in endothelial cells of neo-vessels [51] and along with S100 calcium-binding protein (S100A) they were identified as potential biomarkers of infective endocarditis.

For several found genes, a role has been reported in blood such as (1) colony-stimulating factor 3 receptor (CSF3R) that encodes a receptor protein for colony-stimulating factor 3, a cytokine that regulates the assembly, differentiation, and role of granulocytes [52], (2) cystatin F (CST7) known to be expressed by CD8+ T-cells [53], and (3) Fc fragment of IgA receptor (FCAR) that is a member of the immunoglobulin gene superfamily localized on the surface of, e.g., neutrophils, monocytes, macrophages, and eosinophils, with a role in immunologic responses to pathogens [54]. In particular, using the Reactome pathways, 12 of 27 mRNAs are involved in neutrophil degranulation (i.e., matrix metallopeptidase 9 MMP9, C-X-C motif chemokine receptor 1 CXCR1, formyl peptide receptor 1 FPR1, Selectin L SELL, and leukocyte immunoglobulin-like receptor A5 LILRA5) [55].

Blood lncRNAs have not yet been reported in the literature.

*miR-144*, which correlates positively with Kruppel-like factor 1 (KLF1) and glycophorin A (MNS blood group) (GYPA), regulates hematopoiesis and vascular development by inhibiting expression of MEIS1 [56].

*miR-223*, which correlates positively with 16 mRNAs (APOBEC3A, AQP9, CSF3R, CST7, CXCR1, CXCR2, FFAR2, FPR1, FPR2, LILRA5, NFE2, S100A12, S100A9, SELL, TREML2, and VNN2) and 2 lncRNAs (AOAH-IT1 and AC008984.2), is a highly conserved miRNA [57] linked to granulopoiesis [58].

### 4.5. Brain

TS-co-expression gene regulatory network in the brain constitutes one of the biggest networks with 195 mRNAs, 102 lncRNAs, and 1 miRNA (*miR-3665*) sharing 1512 interactions.

Using the Reactome pathways, 38 of 195 mRNAs are involved in neuronal system (i.e., gamma-aminobutyric acid-type A receptor gamma 1 subunit GABRG1 and 2, glutamate decarboxylase 2, protein kinase C gamma, and G protein subunit gamma 3), 29 of 195 mRNAs in transmission across chemical synapses, and 21 of 195 mRNAs in neurotransmitter receptors and postsynaptic signal.

*miR-3665* is positively correlated with 3 mRNAs (contactin 2, myelin oligodendrocyte glycoprotein, and myelin-associated glycoprotein) and 1 lncRNA (RP11-116N8.1).

### 4.6. Breast

TS-co-expression gene regulatory network in the breast involves 1 mRNA (ANKRD30A) and 2 lncRNAs (RP11-20F24.2 and CTD-3032H12.1), sharing 2 interactions.

Ankyrin repeat domain 30A (ANKRD30A) encodes a transcription factor that is exclusively expressed in the breast and the testis. Deregulated expression levels have been correlated with breast cancer progression [59].

The expression of the locus containing the lncRNA RP11-20F24.2 has been already described in the breast, being altered in triple-negative breast cancer [60], while the lncRNA CTD-3032H12.1 has a role of a proliferation controller in *in vitro* experiment on breast cancer cell lines [61].

### 4.7. Cervix Uteri

TS-co-expression gene regulatory network in the cervix uteri involves 3 mRNAs (HOXA13, MAS1, and TGM6), 3 lncRNAs (RP11-246A10.1, XX-C2158C12.2 and RP11-893F2.15), and 1 miRNA (*miR-4269*), sharing 5 interactions.

Homeobox A13 (HOXA13) is highly expressed in the uterus, and its variation can alter development of the female reproductive tract [62].

For MAS1 proto-oncogene, G protein-coupled receptor (MAS1), transglutaminase 6 (TGM6), RP11-246A10.1, XX-C2158C12.2, RP11-893F2.15, and *miR-4269* has not yet been reported a specific role in the cervix uteri.

### 4.8. Colon

TS-co-expression gene regulatory network in the colon involves 6 mRNAs (CHRM2, HAND1, MAB21L2, PHOX2A, PHOX2B, and PIRT) and 3 lncRNAs (CTB-158E9.1, RP11-227F19.1 and RP11-1336O20.2), sharing 6 interactions. Cholinergic receptor muscarinic 2 (CHRM2), a muscarinic cholinergic receptors belonging to a larger family of G protein-coupled receptors, and mab-21 like 2 (MAB21L2) have not yet reported in colon tissue.

Paired-like homeobox 2A and B (PHOX2A and PHOX2B) are involved in the intestinal neuronal differentiation playing a direct role in the pathogenesis of enteric disorders [63]. The heart and neural crest derivatives expressed 1 (HAND1), belonging to the basic helix-loop-helix family of transcription factors, is showed to be a tumor suppressor gene in colorectal cancer [64]. Phosphoinositide interacting regulator of transient receptor potential channels (PIRT) was already described in various regions of the gastrointestinal tract of the adult mouse [65]. Regarding the lncRNAs, RP11-1336O20.2 is the only lncRNA that has been described in colon cancer tissue [66].

### 4.9. Fallopian Tube

TS-co-expression gene regulatory network in the fallopian tube involves 25 mRNAs, 39 lncRNAs, and 7 miRNAs (*miR-23b*, *miR-369*, *miR-1225*, *miR-3125*, *miR-3613*, *miR-3622a*, and *miR-3671*), sharing 189 interactions.

According to the Reactome pathway, among 25 mRNAs, several are involved in GPCR downstream signaling such as bombesin receptor subtype 3 (BRS3), phosphoinositide-3-kinase regulatory subunit 2 (PIK3R2), and olfactory receptor family 51 subfamily I member 2 (OR51I2), but there are not any mRNAs with a known role in the fallopian tube to date.

Previous studies have shown the role of *miR-23b* in cervical stem cells and ovarian cancer but no study has reported a role in the fallopian tube for *miR-369*, *miR-1225*, *miR-3125*, *miR-3613*, *miR-3622a*, and *miR-3671*, as well as for the 39 lncRNAs [67].

### 4.10. Heart

TS-co-expression gene regulatory network in the heart involves 20 mRNAs (CKMT2, DHRS7C, FBXO40, HRC, METTL11B, MYBPC3, MYH6, MYL3, MYL4, MYL7, MYOM2, MYOZ2, PXDNL, RD3L, SBK2, SMCO1, SMPX, SPHKAP, TBX5, and TNNI3), 11 lncRNAs (AC009878.2, CTD-2194D22.3, KB-173C10.1, RP11-19D2.1, RP11-19O2.1, RP11-213G6.2, RP11-401H2.1, RP11-481J2.2, RP11-532N4.2, RP11-700N1.1, and RP11-774O3.1), sharing 33 interactions.

Creatine kinase, mitochondrial 2 (CKMT2) and dehydrogenase/reductase 7C (DHRS7C) were found highly expressed in the heart [68, 69]. MYH6, MYBPC3, MYL3, MYL4, MYL7, MYOM2, and MYOZ2 encode proteins involved in cardiac function and they may be linked to modulation of cardiac contraction [70, 71].

Peroxidasin-like (PXDNL) was found as a potential peroxidase homologue in the human heart [72]. SH3 domain-binding kinase family member 2 (SBK2) was found expressed in the atrium [37]. Some evidences have shown a role of small muscle protein, X-linked (SMPX), SPHK1 interactor, AKAP domain containing (SPHKAP), T-box 5 (TBX5), and troponin I3, cardiac type (TNNI3) in the heart [73–76].

Retinal degeneration 3-like (RD3L) and single-pass membrane protein with coiled-coil domains 1 (SMCO1) have not yet been reported to have a role in the heart. No publication is present on the role of the 11 lncRNAs in heart tissue.

### 4.11. Kidney

TS-co-expression gene regulatory network in the kidney involves 64 mRNAs, 68 lncRNAs, and 1 miRNA (*miR-1227*), sharing 385 interactions.

According to the Reactome pathway, 24 of 64 mRNAs are involved in transport of small molecules, 17/64 mRNAs in SLC-mediated transmembrane transport, and 9/64 mRNAs in transport of glucose, bile salts and organic acids.

Among mRNAs of the first group, there are solute carrier family, ATPase H + transporting (ATP6V0A4, ATP6V0D2, and ATP6V1G3), and FXYD domain-containing ion transport. No publication is present on the role of *miR-1227* in the kidney.

### 4.12. Liver

TS-co-expression gene regulatory network in the liver involves 133 mRNAs, 44 lncRNAs, and 1 miRNA (*miR-135a1*), sharing 476 interactions.

According to the Reactome pathway, 32 of 133 mRNAs are involved in biological oxidation, 77/133 mRNAs in metabolism, and 29/133 mRNAs in hemostasis.

*miR-135a* has an important role in proliferation and metastatic growth of hepatocellular cells and is associated with malignant thrombosis [77].

### 4.13. Lung

TS-co-expression gene regulatory network in the lung involves 11 mRNAs (ALPP, MS4A15, NAPSA, ROS1, SCGB1A1, SFTA2, SFTPA1, SFTPA2, SFTPB, SFTPC, and SFTPD) and 7 lncRNAs (AC068134.8, CTD-2531D15.4, RP11-312J18.6, RP11-476D10.1, RP11-62I21.1, RP11-95I16.2, and U82670.4), sharing 13 interactions.

Napsin A aspartic peptidase (NAPSA) encodes a member of the peptidase A1 family of aspartic proteases, and it is involved in the processing of pulmonary surfactant protein B in the lung [78]. ROS proto-oncogene 1, receptor tyrosine kinase (ROS1), encodes an orphan receptor tyrosine kinase and is vulnerable to intrachromosomal or interchromosomal rearrangements in several tumor types including non-small-cell-lung cancer [79].

Secretoglobin family 1A member 1 (SCGB1A1) is involved in the regeneration of alveolar epithelia in response to severe pulmonary damage in mice [80]. Surfactant family genes (SFTA2, SFTPA1, SFTPA2, SFTPB, SFTPC, and SFTPD) encode pulmonary surfactant-associated proteins [81]. Alkaline phosphatase, placental (ALPP) and membrane spanning 4-domains A15 (MS4A15) have not yet been reported with a specific role in the lung. None of the 7 lncRNAs has been described in lung tissue before.

### 4.14. Muscle

TS-co-expression gene regulatory network in the muscle involves 38 mRNAs, 12 lncRNAs (CTD-2003C8.2, CTD-2201G16.1, RP11-22P6.3, RP11-293P20.2, RP11-317J10.2, RP11-388M20.9, RP11-567E21.3, RP11-6I2.4, RP11-755O11.2, RP11-882G5.1, RP1-46F2.2 and RP4-597A16.2), and 1 miRNA (*miR-133a-2*), sharing 81 interactions.

Using the Reactome pathway, we found that 20/38 mRNAs (i.e., actinin alpha 2 (ACTN2), myosin heavy chain 13 (MYH13), troponin i1, slow skeletal type (tnni1), calcium voltage-gated channel subunit alpha1 S (CACNA1S), and calsequestrin 1 (CASQ1)) are involved in muscle contraction and 15/38 in striated muscle contraction (i.e., nebulin (NEB) and titin-cap (TCAP)).

*miR-133a-2* along with *miR-133a-1* is muscle-specific miRNAs that is modulated during muscle development by the serum response factor [82]. Although lncRNA RP11-6I2.4 has been recently associated to atherosclerosis [83], no publication is currently available for the identified lncRNAs in the muscle.

### 4.15. Nerve

TS-co-expression gene regulatory network in the nerves involves 4 mRNAs (HCN1, IL9, S100B, and SLC25A48) and 3 lncRNAs (GS1-39E22.2, LINC00462, and PEX5L-AS1), sharing 5 interactions.

Hyperpolarization-activated cyclic nucleotide-gated potassium channel 1 (HCN1) has been found in the central and peripheral nervous system, where they are associated with synaptic integration, neuronal excitability, and formation of resting membrane potentials [84]. Interleukin 9 (IL9), S100 calcium-binding protein B (S100B), and solute carrier family 25 member 48 (SLC25A48) have not yet been reported to have a specific role in the nerves.

### 4.16. Ovary

TS-co-expression gene regulatory network in the ovary involves only 1 mRNA (WFIKKN2) and 1 lncRNA (RP11-506D12.5), sharing only 1 interaction.

WAP, follistatin/kazal, immunoglobulin, kunitz and netrin domain containing 2 (WFIKKN2) has not yet been reported to have a specific role in the ovary.

RP11-506D12.5 along with other 12 lncRNAs was proposed as gene signature to identify breast cancer patients at high risk of tumor recurrence [85].

### 4.17. Pancreas

TS-co-expression gene regulatory network in the pancreas involves 3 mRNAs (IL22RA1, RNF186, and PRSS3) and 3 lncRNAs (RP11-133O22.6, RP11-91K11.2, and RP4-799P18.2), sharing 3 interactions. Interleukin 22 receptor subunit alpha 1 (IL22RA1) and protease serine 3 (PRSS3) were associated with chronic pancreatitis in mouse models [86] and with progression, metastasis, and prognosis of human pancreatic cancer [87].

Ring finger protein 186 (RNF186) has not yet been reported in the pancreas. No publication is found on the role of the 3 identified lncRNAs in the pancreas function.

### 4.18. Pituitary

TS-co-expression gene regulatory network in the pituitary involves 47 mRNAs, 34 lncRNAs, and 4 miRNAs (*miR-7-3*, *miR-7-3hg*, *miR-137hg*, and *miR-770*), sharing 137 interactions. According to the Reactome pathway, 4 of 47 mRNAs are involved in peptide hormone biosynthesis (i.e., glycoprotein hormones, alpha polypeptide (CGA), proopiomelanocortin (POMC), and luteinizing hormone beta polypeptide (LHB)), 3/47 glycoprotein hormones, and 4/47 in class A/1 (rhodopsin-like receptors) (i.e., arginine vasopressin receptor 1B (AVPR1B), and dopamine receptor D2 (DRD2)).

*miR-7* was found to be enriched in the pituitary [88], *miR-137hg* was associated as schizophrenia-associated locus [89], and *miR-770* have been linked to pituitary adenoma [90].

### 4.19. Prostate

TS-co-expression gene regulatory network in the prostate involves 18 mRNAs (ACPP, CHRM1, CHRNA2, HOXB13, KLK2, KLK3, KLK4, LMAN1L, MMP26, NKX3-1, PCAT4, PRAC1, PRAC2, SPDEF, TGM4, TRGJ1, TRGJP, and TRPM8) and 16 lncRNAs (AC005538.5, AC016730.1, AC018804.3, AC092635.1, CTD-2311B13.7, KB-1930G5.4, LSAMP-AS1, PCGEM1, RP11-118K6.2, RP11-366M4.3, RP11-452C8.1, RP11-493K23.4, RP11-536C10.7, RP11-597A11.2, RP11-91I20.3, and RP1-288H2.2), sharing 51 interactions.

Acid phosphatase, prostate (ACPP) is regulated by androgen and is produced by the epithelial cells of the prostate gland [91]. Cholinergic receptors (CHRM1 and CHRNA2), homeobox B13 (HOXB13), matrix metallopeptidase 26 (MMP26), NK3 homeobox 1 (NKX3-1), prostate cancer susceptibility candidate (PRAC1 and PRAC2), SAM pointed domain containing ETS transcription factor (SPDEF), transglutaminase 4 (TGM4), and transient receptor potential cation channel subfamily M member 8 (TRPM8) are associated with prostate cancer [92–99].

Kallikrein related peptidase family (KLK2, KLK3, and KLK4) encode a serine proteases mainly expressed in prostatic tissue. They are highly expressed in prostate tumor cells and may be a prognostic maker for prostate cancer risk [100].

Lectin, mannose-binding 1 like (LMAN1L), and T cell receptor gamma joining family (TRGJ1 and TRGJP) have not yet been associated with the prostate. No publication is currently available on the role of the 16 identified lncRNAs in prostate tissue.

### 4.20. Salivary Gland

TS-co-expression gene regulatory network in the salivary gland involves 28 mRNAs and 23 lncRNAs, sharing 86 interactions.

Polymeric immunoglobulin receptor (PIGR), also known as the membrane secretory component, plays a central role in this network. It has a crucial function in intestinal immunity, but since the oral cavity is protected by the mucosal immune system, PIGR seems to have a role also in the salivary gland [101].

### 4.21. Skin

TS-co-expression gene regulatory network in the skin involves 121 mRNAs, 23 lncRNAs, and 1 miRNA (*miR-205hg*), sharing 711 interactions.

According to the Reactome pathway, 51 of 121 mRNAs are involved in keratinization (i.e., keratin family genes (KRT), desmoglein (DSG), and desmocollin (DSC)), and formation of the cornified envelope (i.e., late cornified envelope 6A and plakophilin).

*miR-205hg* was associated with cutaneous squamous cell carcinoma [102] and controls neonatal expansion of skin stem cells by regulating the PI(3)K pathway [103].

### 4.22. Small Intestine

TS-co-expression gene regulatory network in the small intestine involves 117 mRNAs, 30 lncRNAs, and 4 miRNAs (*miR-147b*, *miR-192*, *miR-194-2*, and *miR-559*), sharing 1197 interactions.

According to the Reactome pathway, 8/117 mRNAs are involved in antimicrobial peptides (i.e., intelectin 2 and regenerating family member 3 alpha and 4), 7/117 mRNAs in O-linked glycosylation of mucins (i.e., mucin 2, 3A, and 17; cell surface-associated and UDP-GlcNAc : betagal beta-1, 3-N-Acetylglucosaminyltransferase 6), 7/117 in MHC class II antigen presentation (i.e., tubulin alpha-like 3 and sucrase-isomaltase), and 5/117 in alpha-defensins (i.e., defensin alpha 5 and 6).

*miR-147b* was found as a biomarker for colorectal tumor, *miR-192* seems to play a vital role in the differentiation and function of the intestinal epithelium, and *miR-194* is regulated by HNF-1 alpha and seems to have important functions in intestinal epithelium maturation [104–106]. To date, *miR-559* has not yet been stated to be associated with the intestine in the literature.

### 4.23. Spleen

TS-co-expression gene regulatory network in the spleen involves 70 mRNAs, 17 lncRNAs, and 1 miRNA (*miR-1587*), sharing 136 interactions.

According to the Reactome pathway, 42/70 mRNAs are involved in FCGR activation (i.e., immunoglobulin lambda constant family and Fc Receptor), 42/70 mRNAs in the role of phospholipids in phagocytosis, 42/70 in Fc gamma receptor- (FCGR-) dependent phagocytosis, and 47/70 in the innate immune system (i.e., integrin subunit alpha D).

*miR-1587* has not yet been reported in the spleen. No publication is currently available on the role of the 17 identified lncRNAs in the spleen.

### 4.24. Stomach

TS-co-expression gene regulatory network in the stomach involves 22 mRNAs (ANXA10, ATP4A, ATP4B, BARX1, CA9, CCKBR, CHIA, CLDN18, CTSE, DPCR1, GIF, GKN2, KCNE2, LHX5, MUC5AC, NCOA7-AS1, PGA4, SLC26A9, SLC9A4, TFF1, TRIM50, and VSIG1) and 10 lncRNAs (AP000320.6, CTD-2331C18.5, HCG21, HNF1A-AS1, RP11-231K24.2, RP11-567G11.1, RP11-708H21.4, RP11-82C23.2, RP4-681L3.2, and RP5-1125M8.2), sharing 37 interactions.

Annexin A10 (ANXA10) is a nuclear protein particularly expressed in the gastric mucosa, and it is a novel marker of gastric differentiation [107].

ATPase H+/K+ transporting subunit family (ATP4A and ATP4B) encodes a proton pump that catalyzes the hydrolysis of ATP. It is also responsible for gastric acid secretion [108].

BARX homeobox 1 (BARX1) regulates robust smooth muscle growth in the stomach by increasing proliferation of myogenic progenitors [109].

Carbonic anhydrase 9 (CA9) plays a role in the metabolism of carbon and has also been associated with hypoxia and proliferation in different cancers such as gastric cancer [110].

Cholecystokinin B receptor (CCKBR), chitinase acidic (CHIA), claudin 18 (CLDN18), and cathepsin E (CTSE) are found mainly in the gastrointestinal tract with high levels in human stomach tissue [111–114].

Gastric intrinsic factor (GIF) and gastrokine 2 (GKN2) encode a glycoprotein secreted by the cells of the gastric mucosa [115, 116]. GIF is necessary for the correct absorption of vitamin B12 [115].

Potassium voltage-gated channel subfamily E regulatory subunit 2 (KCNE2), secreted mucin 5AC, oligomeric mucus/gel-forming (MUC5AC), pepsinogen 4, group I (Pepsinogen A), and trefoil factor 1 (TFF1) are essential for gastric acid secretion and for normal gastrointestinal function [117–120]. Some of them are the main components of the gastrointestinal mucose layer [117–120].

Tripartite motif containing 50 (TRIM50) checks vesicular trafficking for acid secretion in gastric cells [121]. V-set and immunoglobulin domain containing 1 (VSIG1) is stomach-specific for the accurate differentiation of gastric epithelia [122].

Diffuse panbronchiolitis critical region 1 (DPCR1), LIM homeobox 5 (LHX5), and solute carrier family (SLC26A9 and SLC9A4) have not been reported with a specific role in the stomach. No publication is currently available on the role of the 10 identified lncRNAs in the stomach.

### 4.25. Testis

TS-co-expression gene regulatory network in the testis involves 849 mRNAs, 1318 lncRNAs, and 13 miRNAs (*miR-let7dhg*, *miR-181c*, *miR-202*, *miR-371b*, *miR-519a2*, *miR-663a*, *miR-1302-11*, *miR-3150b*, *miR-3180-3*, *miR-3615*, *miR-3943*, *miR-4313*, and *miR-5188*), sharing 184041 interactions.

According to the Reactome pathway, the major part of genes is involved in reproduction, fertilization, sperm motility, and meiosis.

*miR-202* was found in Sertoli cells, and its expression varies between fertile and non-fertile men, suggesting it as a TS biomarker [123].

The other miRNAs have not yet been reported to have a comprehensive role in the testis.

### 4.26. Thyroid

TS-co-expression gene regulatory network in the thyroid involves 4 mRNAs (SLC26A4, KCNJ16, SFTA3, and TCERG1L) and 6 lncRNAs (RP11-7O14.1, AC002539.1, RMST, RP11-896J10.3, RP11-100G15.10, and RP11-462G8.3), sharing 6 interactions.

Solute carrier family 26 member 4 (SLC26A4) has not yet been reported with a clear role in the thyroid, but it is associated with thyroid dysfunction (Pendred's syndrome) [124].

Surfactant-associated 3 (SFTA3), potassium voltage-gated channel subfamily J member 16 (KCNJ16), and transcription elongation regulator 1 like (TCERG1L) have not been associated with the thyroid. Alteration of expression of RMST lncRNA has been described in thyroid cancer tissue [125], while no publication is currently available for the other 5 lncRNAs.

### 4.27. Vagina

TS-co-expression gene regulatory network in the vagina involves 44 mRNAs and 9 lncRNAs, sharing 107 interactions.

According to the Reactome pathway, 25/44 mRNAs are involved in keratinization, formation of the cornified envelope, and developmental biology. No publication is currently available for the 9 lncRNAs.

## 5. Conclusions

Since tissue and cell-type specificity are at the core of human physiology and disease, the comprehension of the genetic underpinnings of complex tissues is fundamental for developing improved diagnostics and therapeutics. Overall, our analysis provides a comprehensive portrait of TS regulatory co-expression networks from thirty different human tissues. This result may be of help in understanding how RNA networks are different in each tissue and ultimately lead us to better understand how miRNAs and lncRNAs can regulate protein coding RNA and TS biological processes.

This work suggests that TS RNA networks need to be analyzed in each tissue in order to understand disease, cellular processes, and potential side effects of drugs inside target tissue.

## Figures and Tables

**Table 1:** Number of samples for each normal tissue from GTEX.

Tissue	# of normal samples
(1) Adipose tissue	577
(2) Adrenal gland	145
(3) Bladder	11
(4) Blood	511
(5) Blood vessel	689
(6) Brain	1259
(7) Breast	214
(8) Cervix uteri	11
(9) Colon	345
(10) Esophagus	686
(11) Fallopian tube	6
(12) Heart	412
(13) Kidney	32
(14) Liver	119
(15) Lung	320
(16) Muscle	430
(17) Nerve	304
(18) Ovary	97
(19) Pancreas	171
(20) Pituitary	103
(21) Prostate	106
(22) Salivary gland	57
(23) Skin	890
(24) Small intestine	88
(25) Spleen	104
(26) Stomach	192
(27) Testis	172
(28) Thyroid	323
(29) Uterus	83
(30) Vagina	96
Total	**8553**

**Table 2:** Number of tissue-specific (TS) miRNAs for each tissue as obtained from the Gini index.

Tissue	TS-miRNAs
(1) Adipose tissue	—
(2) Adrenal gland	*miR-4263*, *miR-1224*, *miR-4709*, *miR-33b*
(3) Bladder	*miR-3972*, *miR-5091*, *miR-93*, *miR-4469*
(4) Blood	*miR-144*, *miR-3909*, *miR-223*
(5) Blood vessel	—
(6) Brain	*miR-3682*, *miR-4477b*, *miR-3665*, *miR-628*
(7) Breast	—
(8) Cervix uteri	*miR-320b2*, *miR-4269*, *miR-548i1*, *miR-593*
(9) Colon	—
(10) Esophagus	*—*
(11) Fallopian tube	*miR-3671*, *miR-3125*, *miR-3622a*, *miR-23b*, *miR-3613*, *miR-369*, *miR-1225*
(12) Heart	—
(13) Kidney	*miR-1227*
(14) Liver	*miR-1295a*, *miR-4783*, *miR-135a1*, *miR-1236*, *miR-4482-1*, *miR-4751*
(15) Lung	*miR-4635*, *miR-4668*
(16) Muscle	*miR-133bhg*, *miR-133b*, *miR-133a2*
(17) Nerve	*miR-378h*, *miR-4500hg*, *miR-5093*, *miR-657*
(18) Ovary	*miR-548I2*, *miR-3960*, *miR-433*, *miR-1257*
(19) Pancreas	—
(20) Pituitary	*miR-137hg*, *miR-339*, *miR-770*, *miR-1179*, *miR-212*, *miR-7-3hg*, *miR-7-3*, *miR-659*
(21) Prostate	*miR-224*
(22) Salivary gland	*miR-642a*
(23) Skin	*miR-205hg*, *miR-567*, *miR-936*, *miR-203*
(24) Small intestine	*miR-559*, *miR-3944*, *miR-192*, *miR-194-2*, *miR-147b*
(25) Spleen	*miR-589*, *miR-378i*, *miR-1587*
(26) Stomach	—
(27) Testis	*miR-1302-11*, *miR-4258*, *miR-4436a*, *miR-1302-3*, *miR-663b*, *miR-219-1*, *miR-3943*, *miR-5707*, *miR-3150b*, *miR-1302-2*, *miR-let7dhg*, *miR-3689b*, *miR-202*, *miR-26a2*, *miR-5188*, *miR-4313*, *miR-3180-3*, *miR-632*, *miR-3615*, *miR-4526*, *miR-181c*, *miR-639*, *miR-519a2*, *miR-371b*, *miR-663a*
(28) Thyroid	*miR-3907*, *miR-135a2*
(29) Uterus	—
(30) Vagina	—
Total	**95**

**Table 3:** Number of tissue-specific (TS) lncRNAs for different tissues.

Tissue	# TS-lncRNAs
(1) Adipose tissue	16
(2) Adrenal gland	42
(3) Bladder	29
(4) Blood	31
(5) Blood vessel	23
(6) Brain	172
(7) Breast	8
(8) Cervix uteri	14
(9) Colon	10
(10) Esophagus	1
(11) Fallopian tube	39
(12) Heart	46
(13) Kidney	116
(14) Liver	108
(15) Lung	34
(16) Muscle	35
(17) Nerve	48
(18) Ovary	32
(19) Pancreas	52
(20) Pituitary	159
(21) Prostate	54
(22) Salivary gland	56
(23) Skin	44
(24) Small intestine	61
(25) Spleen	94
(26) Stomach	14
(27) Testis	3264
(28) Thyroid	53
(29) Uterus	11
(30) Vagina	21
Total	**4687**

**Table 4:** Number of tissue-specific (TS) mRNAs for different tissues.

Tissue	# TS-mRNAs
(1) Adipose tissue	16
(2) Adrenal gland	41
(3) Bladder	28
(4) Blood	101
(5) Blood vessel	4
(6) Brain	260
(7) Breast	4
(8) Cervix uteri	9
(9) Colon	11
(10) Esophagus	2
(11) Fallopian tube	29
(12) Heart	48
(13) Kidney	87
(14) Liver	237
(15) Lung	34
(16) Muscle	127
(17) Nerve	27
(18) Ovary	20
(19) Pancreas	83
(20) Pituitary	155
(21) Prostate	33
(22) Salivary gland	72
(23) Skin	166
(24) Small intestine	144
(25) Spleen	219
(26) Stomach	40
(27) Testis	1563
(28) Thyroid	35
(29) Uterus	6
(30) Vagina	67
Total	**3668**

**Table 5:** Number of co-expression interactions between tissue-specific (TS)-miRNAs, TS- lncRNAs, and TS-mRNAs for different tissues.

Tissue	# of interactions in co-expressed RNAs
(1) Adipose tissue	5
(2) Adrenal gland	6
(3) Bladder	199
(4) Blood	96
(5) Blood vessel	—
(6) Brain	1512
(7) Breast	2
(8) Cervix uteri	5
(9) Colon	6
(10) Esophagus	—
(11) Fallopian tube	189
(12) Heart	33
(13) Kidney	385
(14) Liver	476
(15) Lung	13
(16) Muscle	81
(17) Nerve	5
(18) Ovary	1
(19) Pancreas	3
(20) Pituitary	137
(21) Prostate	51
(22) Salivary gland	86
(23) Skin	711
(24) Small intestine	1197
(25) Spleen	136
(26) Stomach	37
(27) Testis	184041
(28) Thyroid	6
(29) Uterus	—
(30) Vagina	107
Total	**189530**

## Data Availability

The data used to support the findings of this study are included within the article and are derived from the Genotype-Tissue Expression (GTEx) project available at https://gtexportal.org/home/.

## Supplementary Materials

Supplementary Materials Supplementary Table 1: the list of TS-lncRNAs.
